# Research Progress on the Extraction, Structure, and Bioactivities of Polysaccharides from *Coriolus versicolor*

**DOI:** 10.3390/foods11142126

**Published:** 2022-07-18

**Authors:** Yongshuai Jing, Shilin Zhang, Mingsong Li, Yunfeng Ma, Yuguang Zheng, Danshen Zhang, Lanfang Wu

**Affiliations:** 1 College of Chemistry and Pharmaceutical Engineering, Hebei University of Science and Technology, 26 Yuxiang Street, Shijiazhuang 050018, China; cjys1985@126.com (Y.J.); zhangshilin@hebust.edu.cn (S.Z.); limingsong@hebust.edu.cn (M.L.); mayunfeng@hebust.edu.cn (Y.M.); zhangds@hebust.edu.cn (D.Z.); 2 College of Pharmacy, Hebei University of Chinese Medicine, 3 Xingyuan Road, Shijiazhuang 050200, China; zhengyuguang@hebust.edu.cn

**Keywords:** *Coriolus versicolor*, polysaccharides, extraction, structure, bioactivities

## Abstract

Coriolus is the dried fruiting body of *Coriolus versicolor* (L. ex Fr.) Quel. *C. versicolor* (CV) is a worldwide-distributed fungus, which is common and widely used in primitive forests in the northern hemisphere. Polysaccharide, as the main active ingredient in CV, has a variety of biological activities, such as promoting immune function, antivirus, antitumor, anti-diabetes, and so on. However, *Coriolus versicolor* polysaccharide (CVP) faces the problems of a single extraction method, lack of research on separation and purification, and the research on structural characterization is limited to the primary structure. Furthermore, the existing research results have not been systematically reviewed. Therefore, this paper summarizes the research status of CVP in terms of extraction technology, separation and purification, structural characterization, and pharmacological activity in recent years, in order to provide a theoretical basis for in-depth research, development, and utilization of CVP.

## 1. Introduction

*Coriolus versicolor* (CV), also known as *Trametes versicolor* and *Polyporus versicolor*, is a kind of folk medicine and edible fungus growing on tree trunks. CV is mild in nature and sweet in taste. From the perspective of Traditional Chinese Medicine, it enters the heart, spleen, liver, and kidney meridians of the human body and plays important roles in health, such as strengthening the spleen, promoting diuresis, clearing heat, and removing toxicity. It also is used to damp heat jaundice, hypochondriac pain, poor appetite, lassitude, and weakness [1]. At present, many studies have reported that CV has antitumor, antioxidation, hypoglycemic, and immune-enhancing effects, and has potential application value in the treatment of liver disease, diabetes, and other diseases. At the same time, CV is also an important dietary resource. In China, Japan, and the United States, a variety of functional foods and dietary supplements with immunomodulatory activity and liver protection have been developed. The study on the composition of CV shows that the main components of CV are proteins, fats, polysaccharides, polysaccharide peptides, glucans, amino acids, and a variety of inorganic salts [2].

Polysaccharides, as one of the effective components in CV, have many physiological activities, such as promoting immune function, antitumor, anti-inflammation, anti-diabetes, and so on [3,4,5,6]. The extraction of *C. versicolor* polysaccharide (CVP) forms the basis of its in-depth research, development, and application. At present, the extraction methods of CVP mainly include hot water extraction, enzyme extraction, and ultrasonic-assisted extraction [7]. The hot water extraction method is more widely used because of its simple operation, simple equipment, and less damage to the polysaccharide activity. The biological activity of polysaccharides is generally highly dependent on their structural characteristics, which are closely related to physiological functions and play an important role in health [8]. Sun et al. [9] purified six CVP fractions and found that CVP is a polysaccharide with high molecular weight, ranging from 500 to 1000 kDa, and where the main monosaccharide is glucose, followed by small amounts of mannose, rhamnose, glucuronic acid, and fructose. Through methylation analysis and 1D/2D-NMR analysis, Zhang et al. found that its main chain is composed of the (1→4)-β-/(1→3)-β-d-glucopyranosyl group, and branches attached to the O-6 site [10].

**Figure 1:**
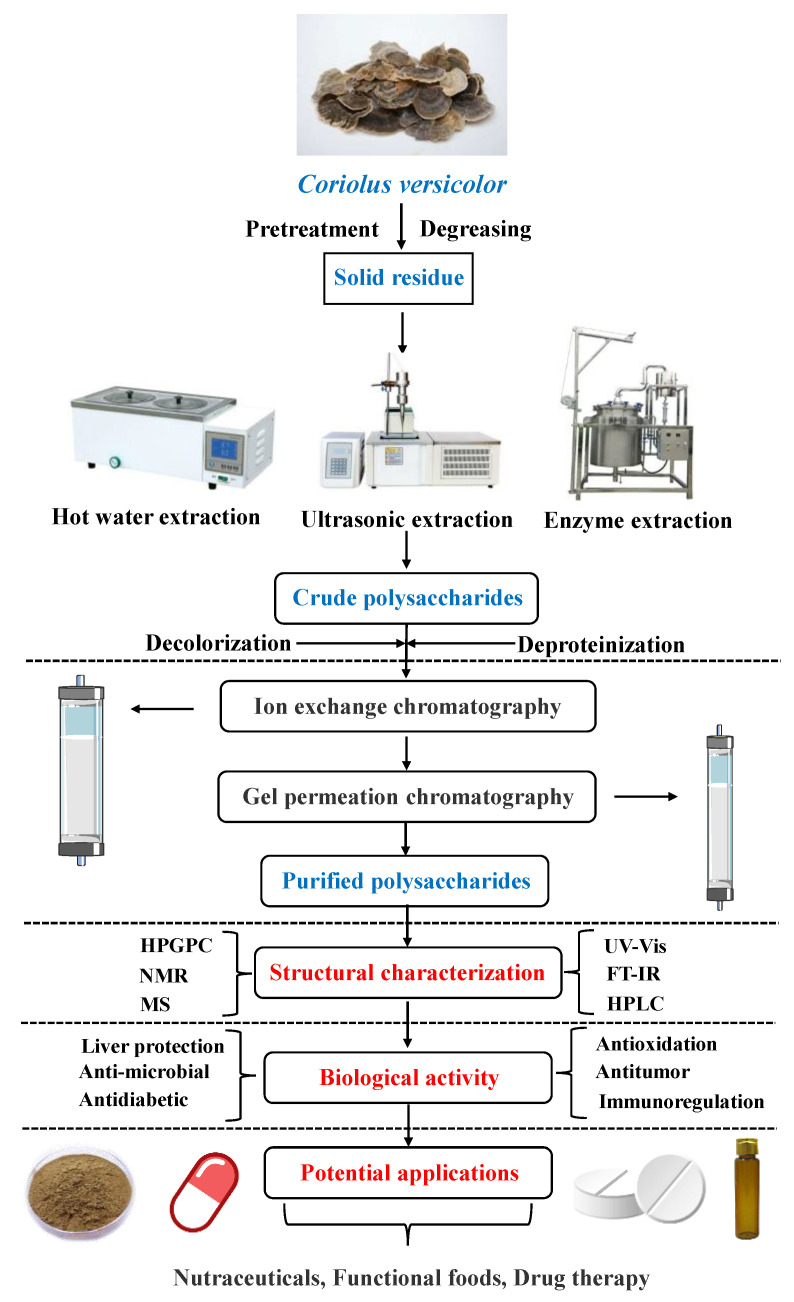
Overall research idea chart of CVP.

At present, CVP faces the problems of a single extraction method, lack of research on separation and purification, and the research on structural characterization is limited to the primary structure. Furthermore, the existing research results have not been systematically reviewed. Therefore, this paper reviews the research status of CVP in recent years from the aspects of extraction technology, separation and purification, structural characterization, and pharmacological activity, in order to provide a theoretical basis for the intensive research, development, and application of CVP. The overall research idea of CVP is shown in Figure 1.

## 2. Extraction of CVP

The main raw materials for extracting polysaccharides from edible and medicinal fungi are fruiting bodies, mycelium, fermentation broth, sclerotia, etc. Fruiting bodies and mycelium are most commonly used [11]. At present, the most commonly used extraction methods are solvent extraction, biological extraction, and physical enhancement extraction [12]. Solvent extraction methods include hot water extraction and alcohol precipitation, acid extraction, alkali extraction, and supercritical fluid extraction. Biological extraction methods include single enzymatic hydrolysis and compound enzymatic hydrolysis. Physically enhanced extraction methods include microwave extraction, ultrasonic extraction, and high-voltage pulse extraction. The extraction rate of CVP is influenced by the extraction method, extraction temperature, and extraction time. Different methods also have their own advantages and disadvantages [13]. Therefore, when extracting CVP, we should choose the appropriate method according to the actual situation. At present, the commonly used methods are hot water extraction, enzyme extraction, and ultrasonic assisted extraction. The extraction method, conditions, and yield of CVP are shown in Table 1.

### 2.1. Hot Water Extraction Method

Polysaccharide contains more hydroxyl groups and other polar substituents, and its solubility in hot water is very large, but its solubility in polar organic solvents, such as ethanol, is very small. Therefore, the main principle of the hot water extraction method is that the external heat makes the cells expand to change the inside and outside osmotic pressure of the cell wall. Through the cumulative action of time and temperature, the polysaccharide is slowly diffused from the cell, and then polar organic solvents such as ethanol are added to precipitate the polysaccharide to finally obtain the polysaccharide [8].

Hot water extraction method mainly includes two processes: hot water extraction and ethanol precipitation. It is worth noting that the material-to-liquid ratio, extraction time, and temperature are the main factors affecting the extraction rate. Generally speaking, within a certain range of changes, a higher extraction temperature and higher extraction time will have a positive impact on the extraction rate of polysaccharides [24]. Previous studies found that the best extraction conditions for hot water extraction of CV mycelium were as follows: a solid-to-liquid ratio of 1:30 (g:mL), extraction temperature of 90 °C, extraction time of 2 h, and then the extraction time twice. Under these conditions, the extraction rate of the mycelial polysaccharide was 5.38% [14]. In contrast, Chen et al. [15] used a higher temperature and longer extraction time for extraction in boiling water, and the average extraction rate of the polysaccharide was about 6.98%, which was higher than 5.38% in the previous study. It can be inferred that temperature and time have a greater impact on the extraction rate of polysaccharide by hot water extraction. Even if the extraction was carried out under the best conditions, the extraction rate of the two might be different, which also showed that the hot water extraction method was affected by many factors. Hu et al. [16] and Sun et al. [17] both carried out response surface methodology optimization experiments based on the material-to-liquid ratio, extraction time, and temperature, and optimized the extraction conditions of CVP. The extraction rates of the two were different, which were 7.27% and 4.39%, respectively. At the same time, the size of the raw material particles will also affect the extraction rate of polysaccharides. The relatively small raw materials particles will increase the contact area with water, which is more conducive to extraction. When the CV medicinal material was crushed by mechanical crushing and liquid nitrogen, and the particle size was smaller than 200 meshes, the extraction rate was significantly higher than that without crushing, which was 16.7% [18].

Hot water extraction is a common extraction method for extracting CVP and has the advantages of simple operation, simple equipment, less damage to the polysaccharide activity, and thus is more suitable for mass production. However, the volume of extraction solvent used in this method is large, the extraction time is long, and the polysaccharide contains numerous hydroxyl groups, which will affect its chemical structure and biological activity at a high temperature for a long time. The experimental process needs to be further improved [25].

### 2.2. Enzyme Extraction Method

The enzyme extraction method is a method to break and degrade the cell wall of Chinese herbal medicine through a mild enzymatic hydrolysis reaction to promote the free release of the effective components [26]. The principle of extracting polysaccharides by this method is that enzymes can selectively and specifically degrade the cell wall and cell membrane of plants, algae, and microorganisms; reduce the resistance from the cell wall, cell membrane, and intercellular substance during solvent extraction; and release the effective components [27,28]. At the same time, the use of enzymes can also degrade some polysaccharides into fragments with smaller molecular weight, which is more conducive to the separation of polysaccharides from cells [29]. CV is a medicinal fungus, and its cell wall is mainly composed of cellulose, protein, and pectin. Therefore, the enzymes used in the enzymatic extraction of CVP are mainly cellulase, various proteases, and pectinase.

The extraction efficiency of the enzyme extraction method also depends on the type and concentration of enzyme, temperature, pH value, reaction time, as well as liquid-to-solid ratio [30]. Yang et al. [19] optimized the compound enzyme extraction process of CVP. The results showed that the best extraction conditions for enzymatic hydrolysis are pH 5.5, enzymolysis time of 37 min, enzymolysis temperature of 52 °C, and enzyme concentration of 2.50%. The extraction rate of enzymatic hydrolysis was 9.58%, which was 43.63% higher than that of traditional hot water extraction. It could be seen that enzymatic hydrolysis has a better extraction effect than the traditional water extraction and alcohol precipitation method, but its experimental operation and condition control are also more complex. Generally speaking, in the range of enzyme activity, higher enzyme concentration, temperature, and longer reaction time will have a positive impact on the extraction rate. Compared with the above, Wang et al. [20] adopted a shorter extraction time, lower concentration, and lower temperature, and the extraction rate of the polysaccharide was only 3.26%, which was far less than that of Yang et al. It can be seen that time, concentration, and temperature are important factors affecting the enzyme extraction method.

In conclusion, the enzymatic extraction of CVP can improve the extraction rate and greatly reduce the extraction time. The extraction conditions of the enzyme extraction method are relatively mild, and the spatial structure of the CVP is less damaged. However, the enzyme extraction method also has some shortcomings. For example, since the enzyme itself is a protein, the enzyme extraction method may increase the protein content in the extracted polysaccharide sample. In the follow-up, the polysaccharide should be separated and purified to remove the protein in the crude polysaccharide. Moreover, the nature of the enzyme is unstable, it is easy to inactivate and difficult to preserve under bad conditions, and the price is also more expensive [31]. Therefore, in the process of enzymatic extraction, the extraction conditions should be strictly controlled to avoid enzyme inactivation or denaturation.

### 2.3. Ultrasonic Extraction Method

Ultrasonic extraction method uses the cavitation effect caused by the formation of acoustic cavitation in solvent and the collapse of asymmetric micro bubbles. These bubbles can release a lot of energy and produce micro jets, shock waves, and high shear forces. Fluid dynamics can promote cell wall rupture, mass transfer between immiscible phases, enhance permeability and capillary effect, and reduce particle size to improve the extraction rate and efficiency [32,33]. Ultrasonic extraction is a universal, low-cost, and easy-to-operate extraction method, which has been applied in the extraction process of CVP.

Studies showed that parameters such as ultrasonic power and frequency, the liquid-to-solid ratio, extraction temperature, and time significantly affect polysaccharide extraction by the ultrasonic extraction method. Previous studies reported that the best ultrasonic extraction process of CVP was 20 times the volume of water, ultrasonic extraction twice for 15 min each time, and the extraction rate was 3.84%. Compared with the hot water extraction method, the extraction rate of the ultrasonic extraction method was not significantly improved, but the extraction time was shortened by nearly four times and the cost was greatly reduced [21]. The results of other studies showed that the optimum extraction conditions were a 2% Na_2_CO_3_ solution as extraction medium, temperature 45 °C, pH 8.0, 35 min, and the extraction rate was 13.87% [22]. Extraction time was an important factor in ultrasonic-assisted extraction. A relatively longer time could improve the extraction rate of CVP, but a too long time might lead to structural damage and degradation of CVP. Ji et al. [23] optimized the extraction conditions of ultrasonic extraction of CVP by the response surface method, and determined that the best operating conditions were an ultrasonic extraction time of 30 min, solid-to-liquid ratio of 1:45 (W:V), and ultrasonic power of 450 W, where the extraction rate could reach 13.6%, which is lower than 13.87%.

The above results show that ultrasonic-assisted extraction has the advantages of low temperature, short time, and a high extraction rate. It is an efficient and green polysaccharide extraction technology. However, the high-intensity shock wave produced by ultrasonic cavitation destroys the integrity of the cell wall of CV, which may have a certain impact on the structure of the polysaccharides [34]. The polysaccharide will change with its degradation during the process of ultrasonic-assisted extraction. Therefore, some measures to inhibit the degradation of the polysaccharides should be taken when using ultrasonic-assisted extraction.

To sum up, the current research on CVP extraction methods is not comprehensive, mainly including hot water extraction, enzyme extraction, and ultrasonic-assisted extraction, and each has its own characteristics. However, there are some problems, such as the research scope is not wide enough, the applicable scene is limited, and there is only a single extraction method. Nowadays, with the continuous development of polysaccharide extraction technology, some new extraction technologies came into being, such as supercritical fluid extraction, microwave-assisted extraction, and ultrafiltration extraction. These new methods can be used to extract CVP to make up for the shortcomings of the existing methods. Therefore, the extraction method of CVP needs further research and development to provide a material basis for the separation, purification, and structural characterization of CVP.

## 3. Isolation, Purification, and Structural Characterization of CVP

The extracted crude polysaccharide will contain protein, pigment, and other active components. Therefore, the extracted polysaccharide needs to be separated, purified, deproteinized, and decolorized. At present, the separation and purification methods of the polysaccharides mainly include ethanol fractionation, column chromatography, and ion exchange chromatography [35]. At the same time, the structure of the polysaccharide is complex and has a multi-level structure. Therefore, many methods need to be used to analyze the structure of polysaccharides at the same time. Structural methods to analyze polysaccharides are generally divided into chemical analysis methods and instrumental analysis methods. Chemical analysis methods include periodate oxidation, methylation reaction, Smith degradation, etc. Instrumental analysis methods include ultraviolet spectroscopy, infrared spectroscopy, high-performance liquid chromatography, nuclear magnetic resonance analysis, and mass spectrometry [36].

The pharmacological activities of CVP are closely related to its structural characteristics, mainly including the type, quantity, and sequence of monosaccharides, as well as the type and spatial concentration of glycosidic bonds. Therefore, the study of the structural characteristics of CVP is helpful to know the pharmacological function of polysaccharides [37]. Xie et al. [38] established a simple and sensitive method for analyzing the monosaccharide composition of CVP. The mobile phase was acetonitrile and 25 mm dipotassium hydrogen phosphate (pH 6.5), using a phenyl kinetex C18 column, and the column temperature was set to 35 °C. The results showed that the monosaccharide composition of CVP included mannose (Man), glucose (Glc), galactose (Gal), and polycaramel (Fuc), with the relative molar ratio being 2.71:63.80:3.53:1.18. This showed that SMP was a neutral polysaccharide with Glc as the main monosaccharide. Due to its high glucose content, it was speculated that SMP might have good antioxidant activity and probiotic activity. In another study, the Fourier transform infrared spectroscopy (FT-IR) spectra of CVP showed its structure in the range of 4000–400 cm^−1^. Polysaccharides with different structures and contents were detected in the mid infrared band of 800–1200 cm^−1^, mainly from the C–C and C–O stretching vibrations of the glycosidic bonds and pyranose rings. In addition, it was found at 1024 cm^−1^ for the β-glycosidic bond and β-glucan and at 920–930cm^−1^ for the α-glycosidic bond [39]. According to the above two studies, CVP might have β-d-Glc*p*, which was similar to the structure of immune active polysaccharide β-d-glucan, suggesting that CVP had good immunomodulatory activity. Wang et al. [40] obtained polysaccharopeptide (PSP) component PSP-1-3 by the stepwise alcohol precipitation method, and the polysaccharide part occupied the main part. The PSP-1b1 was isolated and purified from PSP-1b by gel permeation chromatography. PSP-1b1 is a homogeneous polysaccharide with a molecular weight of 21.7 kDa, and is composed of polycaramel, Gal, Xyl, Man, and Glc, with a relative molar ratio of 0.16:0.60:0.02:0.55:0.04:1.00. Glc was its main monosaccharide, which was basically consistent with the results of Wang et al. Nuclear magnetic resonance spectroscopy (NMR) analysis showed that the main chain of PSP-1b1 changed from →1-β-d-Gal*p*-(6→1)-β-d-Glc*p*-(4→1)-α-d-Gal*p*-(3→1)-α-d-Man*p*-(2→1)-β-d-Glc*p*-4→residues. The results also showed that CVP had β-d-Glc*p*, which was consistent with the above speculation. In fact, the variety, processing technology, and extraction method of CVP had effects on the monosaccharide type, molecular weight, and chemical composition structure of CVP. Another study also revealed that the average molecular weight of PSP was 1.61 and 41.7 kDa, respectively. It is composed of Ara, Fuc, Xyl, Man, Glc, and Gal, and the molar ratio was 0.008:0.005:0.012:0.045:1:0.045. The total sugar content and protein content of PSP were 38.59% and 34.55%, respectively [41].

**Figure 2:**
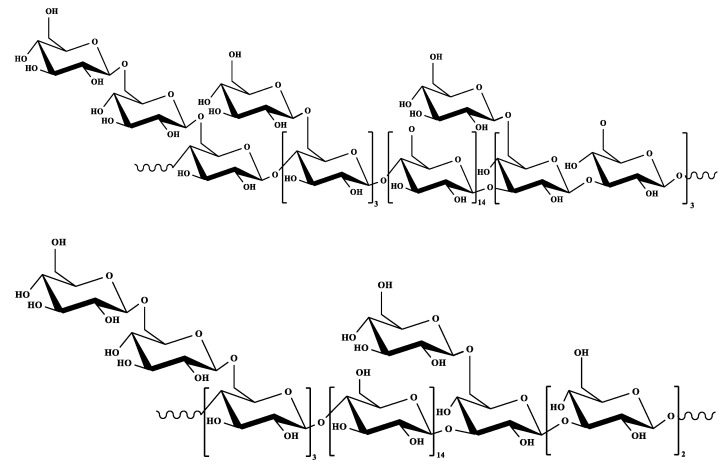
Two possible spatial speculative structures of CVP.

Therefore, CVP is a high molecular weight polysaccharide, Glc is the main monosaccharide, and also contains a small amount of Man, Rha, Gal, and Fuc. Its main chain is composed of the (1→4)-β-/(1→3)-β-d-glucopyranosyl group, and branches attached to the O-6 site. The speculative structure of CVP is shown in Figure 2. The study on the structural characterization of CVP will help us understand and master the physiological activity of CVP and provide a theoretical basis for the study of the physiological activity of CVP. At present, researchers are mostly limited to the study of primary structures, such as functional groups, monosaccharide composition, and the glycosidic linkage mode of CVP, while there are few studies on the higher-order structures of polysaccharides, and few reports on the isolation and purification of CVP. Therefore, the detailed structural elucidation of CVP needs further investigation.

## 4. Bioactivities of CVP

**Figure 3:**
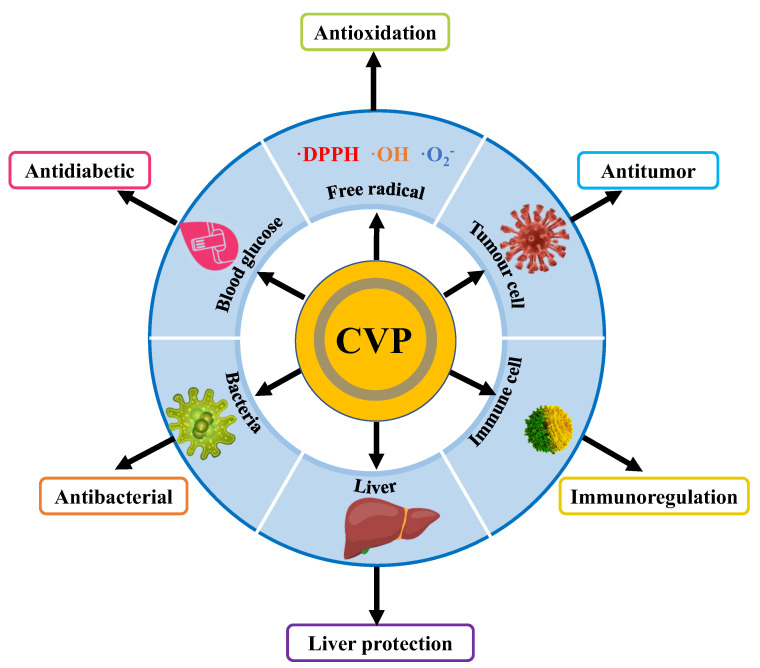
Schematic diagram showing various biological activities of CVP.

CVP is one of the important bioactive substances in CV and it has a variety of pharmacological activities, including antioxidation, antitumor, immune regulation, liver protection, repair of cerebral ischemia–reperfusion injury, and anti-diabetes. As a macromolecular polysaccharide, CVP has a complex spatial structure, and its biological activities are often closely related to its structure. Generally speaking, it mainly includes molecular weight, monosaccharide type, and polysaccharide conformation. In order to promote the product development and clinical application of CVP, we systematically review the biological and physiological activities of CVP and preliminarily discussed its structure–activity relationship to provide reference for the development and utilization of polysaccharide drugs and health products. The various biological activities of CVP are shown in Figure 3.

### 4.1. Antioxidation

Under normal circumstances, the human body will produce a certain amount of free radicals to regulate cell growth and inhibit viruses and bacteria. However, excessive production of free radicals can also lead to various types of illness, such as arteriosclerosis, Alzheimer′s disease, and cardiovascular and cerebrovascular diseases [42]. As an antioxidant, polysaccharides can prevent and treat these diseases by breaking the peroxide chain reaction. Numerous studies have shown that polysaccharides can scavenge free reactive oxygen species, and their groups can combine with hydroxyl radicals or superoxide anion radicals to form water to eliminate free radicals. As an active component of polysaccharide categories, CVP also has good antioxidant activity, and is an excellent biological antioxidant with broad development prospects. The experimental methods and research results are shown in Table 2.

Many studies have found that the monosaccharide composition (glucuronic acid, galactose, and glucose) and molecular weight had a great impact on the antioxidant activity of polysaccharides in vitro [50]. The content of glucuronic acid (GlcA) in acidic polysaccharides was positively correlated with its antioxidant activity, and the higher the content of GlcA, the better the antioxidant activity. In neutral polysaccharides, the higher the content of glucose, the better the antioxidant activity. The smaller the molecular weight, the more dispersed the state, which is conducive to the combination with oxygen free radicals, and the stronger the antioxidant activity [51]. The monosaccharide composition of CVP included Man, Glc, Gal, and Fuc, and the relative molar ratio was 2.71:63.80:3.53:1.18. and the molecular weight was 1.61 kDa and 41.7 kDa. It can be seen that CVP contains a lot of Glc and has a small molecular weight, so it is speculated that CVP has good antioxidant activity. Sun et al. [9] reported CVPS-3 exhibited higher DPPH radical scavenging activity (64.9% at 0.8 mg/mL), superoxide radical scavenging activity (78.4% at 1.2 mg/mL), and hydroxyl radical scavenging activity (71.2% at 2.0 mg/mL), which is slightly less than about 90% of Vc at the same concentration, showing certain antioxidant activity. Another study measured the ability of CVP to scavenge the 1,1-diphenyl-2-picrylhydrazyl (DPPH) radical, 2,2′-azino-bis (3-ethylbenzothiazoline-6-sulfonic acid) (ABTS) radical, and OH radical. The results showed that at 0.8 mg/mL, 0.6 mg/mL, and 7.0 mg/mL CVP had the maximum scavenging rates of the three free radicals, which were all about 85%, while at the same concentration, ascorbic acid (Vc) had the scavenging capacity of about 95%. The scavenging capacity of CVP was close to that of Vc, showing a slightly stronger antioxidant activity [43]. The above two results showed that CVP had good antioxidant activity, which confirmed the above speculation. Wang et al. [44] reported that the total fermentation products of CV and their polysaccharides have obvious scavenging ability against the superoxide anion radical (O_2•_^−^) and hydroxyl (OH) radical. The results showed that at 5 mg/mL, the scavenging rates of CVP for two kinds of free radicals were 60% and 81%, respectively, which is slightly less than 95% of Vc at the same concentration, showing a slightly stronger antioxidant activity. At the same time, the antioxidant activity of CVP might be closely related to the type, origin, and growth environment of CV. Sun et al. [45] found that CVP has a different DPPH radical scavenging capacity in different regions. The results showed that the scavenging capacity of CVP against the DPPH radical in Heilongjiang was the best, and the IC_50_ was 0.832 g/mL. Chai et al. [46] studied the antioxidant activity of polysaccharides extracted from wild CV and cultivated CV. The results showed that the scavenging rates of the O_2•_^−^ and OH radical of the wild varieties at the maximum concentration were 53% and 72%, respectively, which were significantly higher than those of the cultivated varieties (*p* < 0.05). Some previous antioxidant experiments in vivo have explored the antioxidant effect of CVP on rat brain and its mechanism. The results showed that CVP could increase the activity of superoxide dismutase (SOD) and glutathione peroxidase (GSH-Px), as well as reduce the content of malondialdehyde (MDA), promoted free radical scavenging, and have an obvious antioxidant effect [47]. Fang et al. [48] took mice as the research object and measured the expression levels of SOD and catalase (CAT) in the mouse brain. After CVP participated in the treatment, the expression levels of the two enzymes increased and the oxidation level in the brain decreased, indicating that CVP had good antioxidant activity in vivo. In the antioxidant experiment in mice, researchers found that the glycopeptide of CV (PSK) could reduce the oxidative damage of oxidized low-density lipoprotein (Ox-LDL) to macrophages, improve the SOD activity of mouse peritoneal macrophages, and increase the content of manganese superoxide dismutase (MnSOD) mRNA [49].

To sum up, Vc, as an excellent antioxidant, has strong free radical scavenging ability, up to about 95%. Although the scavenging capacity of CVP is mainly 60–90%, slightly lower than that of Vc, it also shows that CVP has strong antioxidant activity, which may be attributed to its relatively high Glc content and small molecular weight, which is consistent with the structure–activity relationship analysis. The results of the antioxidant experiment in vitro show that CVP has good scavenging activity against the DPPH radical, OH radical, and ABTS radical. The results of the antioxidant experiment in vivo showed that CVP can increase the activity of SOD, GSH-PX, and CAT, reduce the content of MDA, accelerate the elimination of free radicals, and have an obvious antioxidant effect. At present, the antioxidant research of CVP mainly focuses on in vitro experiments, but the research on the effect and mechanism of antioxidants in vivo is still insufficient, so it is necessary to strengthen the research in this field.

### 4.2. Antitumor Activity

Cancer, also known as a malignant tumor, is a disease caused by the abnormal mechanism of cell growth and proliferation. It has become a major threat to human life and health. Chemotherapy and antitumor drugs are commonly used at present, but they also show serious side effects. Therefore, it is very important to find new alternative anticancer drugs. A large number of studies have reported that natural polysaccharides have shown good anticancer activity in in vitro and in vivo animal studies, with less toxic side effects, providing a new direction for cancer treatment [52,53]. Therefore, it is of great significance to study the antitumor activity and mechanism of CVP. The experimental model and action mechanism are shown in Table 3.

Most of the results showed that most polysaccharides with β-(1→3)-d-glucan as the main chain had antitumor activity. In addition to glucan, some exoglycans and galactans also show antitumor activities to varying degrees. In addition, it was found that some heteropolysaccharides also have antitumor activity [54,55]. The branching degree of the molecular structure of plant polysaccharides is closely related to the antitumor activity. Different branching degrees show different biological activities. The above structural analysis results shown that the main chain of CVP was composed of the (1→4)-β-/(1→3)-β-d-glucopyranosyl group, and its branches were attached to the O-6 site, with molecular weights of 1.61 kDa and 41.7 kDa, respectively, indicating that CVP had good antitumor potential. Some studies had shown that the antitumor mechanism of CVP might be related to the downregulation of the Bcl-2 and Fas gene expression in cells. Wei et al. [56] studied the effect and mechanism of proliferation and apoptosis of mouse melanoma B16 cells in vitro and found that CVP could inhibit the proliferation and induce apoptosis of mouse B16 cells, and its mechanism might be related to downregulating the expression of P53, Bcl-2, and Fas genes in cells. At the same time, they also found that CVP could inhibit the growth and induce apoptosis of HeLa cells, and its mechanism was related to the down regulation of Bcl-2 gene expression [57]. Regulating the release of immune factors was also an important mechanism of CVP’s antitumor activity. Sun et al. [58] and Ge et al. [59] reported that CVP could promote the proliferation of NK cells and the release of a variety of immune factors through immune regulation to inhibit the proliferation of tumor cells and realize the apoptosis of tumor cells. The antitumor activity of CVP might be related to its molecular weight. The appropriate molecular weight was easier to combine with the receptors on the surface of tumor cells, thus affecting the proliferation of tumor cells. A study found that CVPs-b with a molecular weight of 39,000 Da could inhibit the proliferation and apoptosis of esophageal carcinoma (ESCA) cells, regulate the cell cycle of ESCA cells, and induce more tumor cells to enter a quiescent state faster [60]. CVP could also be combined with XELOX to treat advanced colorectal cancer, which can improve the long-term efficacy and has clinical value [61]. Previous studies showed that CVP also has antitumor and anti-metastatic effects on mouse breast cancer 4T1 cells and 4T1 tumor mice [62]. All the above results showed that CVP had a certain antitumor activity, proving the correctness of the above speculation. Polysaccharide peptide (PSP) was also cytotoxic to HL-60 cells, and its mechanism was speculated to induce apoptosis through upregulation of early transcription factors such as AP-1 and EGR1 [63]. PSP could inhibit the proliferation and induce apoptosis of HL-60 and U-937 cells and destroy the G1/s and G2/M phases of the cell cycle, indicating that PSP had a good antitumor effect [64]. Meanwhile, Wan et al. [65] also reported that PSP could inhibit cell proliferation, inhibit the progress of cells in the S phase and G2 phase, reduce the uptake of ^3^H thymidine in HL-60 cells, and prolong the time of DNA synthesis to inhibit tumor growth.

The above studies show that CVP has good antitumor activity, which is related to its main chain and the appropriate molecular weight, which are conducive to its binding with cell sites and play a role in health. On the one hand, CVP directly acts on tumor cells, affects the s and G2 phases of the cell cycle of tumor cells, upregulates the early transcription factors, induces apoptosis or inhibits oncogene expression, and directly kills tumors. On the other hand, it can enhance the immune ability of the body by acting on the inflammatory factor pathway CXCL12/CXCR4 and promote the growth of NK cells to kill tumor cells. Therefore, CVP has less-toxic side effects and a good antitumor effect. It is expected to become a new antitumor drug. However, the detailed mechanism of CVP killing tumor cells is not clear, and further research is needed.

**Table 3:** Antitumor effects of CVP and its presumed mechanism.

Cell/Animal	Effect	Speculative Mechanism	Ref.
Mouse melanoma B16 cells	↓	P53↓, Bcl-2↓ and Fas↓	[56]
Human cervical cancer HeLa cells	↓	Bcl-2↓	[57]
Human NK cells cultured in vitro	↓	NKG2Dreceptor↑	[58]
H_22_ liver cancer transplanted mice	↓	Its mechanism may might be related to immune regulation and promoting tumor cell apoptosis	[59]
Human esophageal cancer cell line Eca-109	↓	It acted on the inflammatory factor pathway CXCL12/CXCR4	[60]
Colorectal cancer cells	↑	CVP could improve the long-term efficacy in the treatment of patients with advanced colorectal cancer	[61]
Mouse breast cancer 4T1 cells	↓	It has had antitumor and anti-metastatic effects on murine breast cancer 4T1 cells and 4T1 tumor mice	[62]
Human promyelocytic leukemia HL-60 cells	↓	AP-1↑, EGR1↑	[63]
Leukemia HL-60 and U-937 cells	↓	PSP could disrupt the G1/S phase and G2/M phase during the cell cycle	[64]
Human promyelocytic leukemia HL-60 cells	↓	^3^H thymidine↓	[65]

Notes: ↑, Increased; ↓, reduced; 4T1, breast cancer cells; AP-1, activator protein 1; Bcl-2, B-cell lymphoma 2; CXCL12, chemokines; CXCR4, chemokine receptor; EGR1, early growth response 1; Eca-109, human esophageal cancer cells; HL-60 cells, human promyelocytic leukemia cells; NK, natural killer cell; NKG2D, natural killer group 2 member D; P53, tumor suppressor gene; U-937 cells, human leukemia cell.

### 4.3. Immunoregulation Activity

Disease prevention and treatment is the most important goal to enhance the body′s resistance through exercise and therapies related to improving immunity. The vast majority of polysaccharides have immunomodulatory effects [66], and a variety of pharmacological actions of polysaccharides seem to be related to their immunomodulatory actions. This may be because polysaccharides are one of the immune system′s primary targets. T lymphocytes, B lymphocytes, natural killer cells, and macrophages are important immune cells, which play a crucial role in the immune system through cellular immunity [39,67]. As a natural polysaccharide, CVP also has the function of immune regulation. It can be used as an immune regulator or biological response regulator [68]. Animal models and regulatory effects are shown in Table 4.

The low toxicity, high molecular weight, branching structure, and conformation of polysaccharides may be important for the host’s immune system to produce appropriate responses. In different types of polysaccharides, β-d-glucan is considered to be the most important immunomodulatory polysaccharide [69]. β-d-glucan is a repeating structure of the d-glucose unit, which is expressed by β-glycosidic bonds that are linked together. So far, several straight and branched chains with great biological activity potential have been reported for β-d-glucan [70,71]. According to the previous analysis of CVP structure, the main chain of CVP is composed of the (1→4)-β-/(1→3)-β-d-glucopyranosyl group, and glucose has the highest content of monosaccharides, indicating that it has good immunomodulatory potential. Li et al. [72] found that CVP could effectively improve the neural function in a rat cerebral ischemia–reperfusion injury (Ciri) model through immune regulation and the mechanism is related to inhibiting the activation of the p38MAPK signaling pathway. CVP could also significantly induce the upregulation of CD80, CD86, and MHCII molecules expressed by bone marrow-derived dendritic cells, and stimulate BMDC cells to release IL-6, IL-12p40, and TFN-α to realize immune regulation [73]. These results showed that CVP had certain immunomodulatory activity, which proved the correctness of the above speculation. Pramudya et al. [74] and Awadasseid et al. [75] also reported that CVP could restore the number of leukocytes and improve the level of antibody, TNF-α, and IFN-γ, reduce the phagocytic activity and nitric oxide (NO) level, and prevent the overexpression of proinflammatory cytokines. Therefore, CVP can be used as an effective compound to regulate immune response. At the same time, the synthesis of NO and related enzymes were closely related to the immunomodulatory activity of CVP. Kang et al. [76] reported that the phagocytosis activated by CVP was mediated by Syk- and CK2-dependent signaling pathways, and also confirmed that CVP stimulates phagocytosis by activating endothelial nitric oxide synthase (eNOS), inducible nitric oxide synthase (iNOS), and TNF-Dectin-1 signaling pathways, which helps to clarify the mechanism of glucan-induced phagocytosis. CVP could also restore the spleen and thymus index, enhance the proliferation of T cells and B cells and phagocytosis of peritoneal macrophages in immunosuppressive mice induced by cyclophosphamide. In a study, CVP might improve immune function by enhancing the proliferative activity of immune cells, inducing immune cells to produce NO, regulating or promoting the secretion of cytokines [77]. Similarly, previous studies also showed that *Coriolus versicolor* β-Glucan (CVG) increased the expression of SR-B1 by activating the CK2 pathway and Dectin-1 signal. The phagocytic activity of CVG was stronger than that of microorganisms and algae [78]. In in vivo antitumor experiments in mice, the researchers found that after CVP stimulation, the phosphorylation levels of ERK-1/2 and p38MAPK increased significantly in a time-dependent manner. CVP could use the cell membrane Ig and TLR-4 as potential immune receptors to activate mouse B cells through the MAPK and NF-κB signaling pathways [79]. In future research, researchers can have a more in-depth discussion.

These results show that CVP has good immunoregulatory activity, which is related to its (1→4)-β-/(1→3)-β-d-glucopyranosyl groups, and a high content of glucan is closely related. On the one hand, the immunomodulatory activity of CVP depends on the role of NK cells, B cells, T cells, and macrophages. On the other hand, it regulates the number of leukocytes, interleukin, TNF-α, IFN-γ, and other cytokines, and stimulates the TLR4, MAPKs to NF-κB, and other pathways. It can be seen that CVP is a polysaccharide with multiple immunomodulatory functions, which is expected to be developed into a new immunomodulator, but its specific regulatory mechanism and safety need to be further investigated.

**Table 4:** Immunomodulatory effect of CVP.

Animal Model	Regulating Effect	Ref.
Sprague-Dawley rat model of cerebral ischemia-reperfusion injury	P-P38MAPK and caspase-3↓	[72]
Bone marrow cells from C57BL/6 mice	CD80 and CD86↑, IL-6, IL-12p40 and TFN-α↑	[73]
BALB/C mice infected with *N. gonorrhoeae*	TNF-α and IFN-γ↑, NO↓	[74]
Sarcoma-180 tumor bearing C57BLkunmingmice	IL-2, -4, -6, -10, -17A, and IFN-α, -γ↑	[75]
RAW264. 7 mouse monocyte/macrophage line	eNOS, iNOS, and TNF-α↑	[76]
ConA- or LPS-induced Kunming mice	Immune cells and cytokines↑	[77]
RAW264. 7 mouse monocyte/macrophage line	SR-B1↑, Dectin-1↑, CK2↑	[78]
Female BALB/c, C3H/HeJ, C3H/HeN mice	Membrane Ig and TLR4↑	[79]

Notes: ↑, Increased in quantity or expression; ↓, reduced in quantity or expression; CD80, cluster of differentiation 80; CD86, cluster of differentiation 86; COA, concanavalin A; eNOS, endothelial nitric oxide synthase; IL-2, interleukin-2; IL-4, interleukin-4; IL-6, interleukin-6; IL-10, interleukin-10; IL-17, interleukin-17; IL-12p40, interleukin-12p40; INF-α, interferon α; INF-γ, interferon γ; iNOS, inducible nitric oxide synthase; LPS, lipopolysaccharide; NO, nitric oxide; p38 MAPK, p38 mitogen-activated protein kinase; SR-B1, high density lipoprotein receptor; TNF-α, tumor necrosis factor-α; TLR4, toll-like receptor 4.

### 4.4. Other Activities

Besides the above physiological activities, CVP also plays an important role in protecting the liver, antibacterial effects, cerebral ischemia–reperfusion, and treatment of diabetes. Wang et al. [80] studied the hepatoprotective effect of CVP by establishing the NIAAA alcohol injury mouse model. The study found that CVP could significantly reduce the levels of alanine aminotransferase (ALT), aspartate aminotransferase (AST), total cholesterol (TC), glutamine transaminase (TG), and other enzymes, and increase the level of high-density lipoprotein, which played a role in protecting the liver. In another study, CVP was stable at room temperature, had antibacterial action against both Gram-positive and Gram-negative bacteria, and had a broad spectrum of antibacterial action. It was speculated that the polysaccharide macromolecule had good surface activity, which could promote the dissolution of the outer wall of harmful bacteria and destroy the survival and reproduction of harmful bacteria [81]. At the same time, CVP also could improve the behavioral ability of Ciri rats, reduce the volume of cerebral infarction, inhibit neuronal apoptosis, and had a good protective effect on nerve injury after cerebral ischemia–reperfusion [72]. Wang et al. [82] reported that the CV extract with a polysaccharide content of 17.74% (CV/W) could significantly reduce cardiac fibrosis in diabetes rats and inhibit transforming growth factor β1(TGF-β1)/Smad signal transduction, and could also significantly reduce cardiac inflammation in diabetes rats, significantly improving cardiac dysfunction.

## 5. Product Development of CVP

As an important component with low toxicity and significant biological activity, polysaccharides play an important role in medical treatment and biological health care. More and more natural polysaccharides have been developed for modern food supplements and clinical drugs [83]. Due to the above physiological activities, CVP is often used in the fields of health products, functional foods, and drugs. In the Chinese market, there are 156 drugs named CV and functional foods approved by the state administration of market supervision. CVP health products mainly have the effects of protecting the liver, improving immunity, and postoperative cure. In the field of drug treatment, it is mainly made into tablets, capsules, granules, and oral liquids to help treat chronic hepatitis B, liver cancer, and other diseases. At present, the drug application of CVP mainly focuses on liver protection and treatment. The application of other activities still has great development potential, and the development of health products is less, so it needs to be further expanded in the field of health products and drugs.

**Figure 4:**
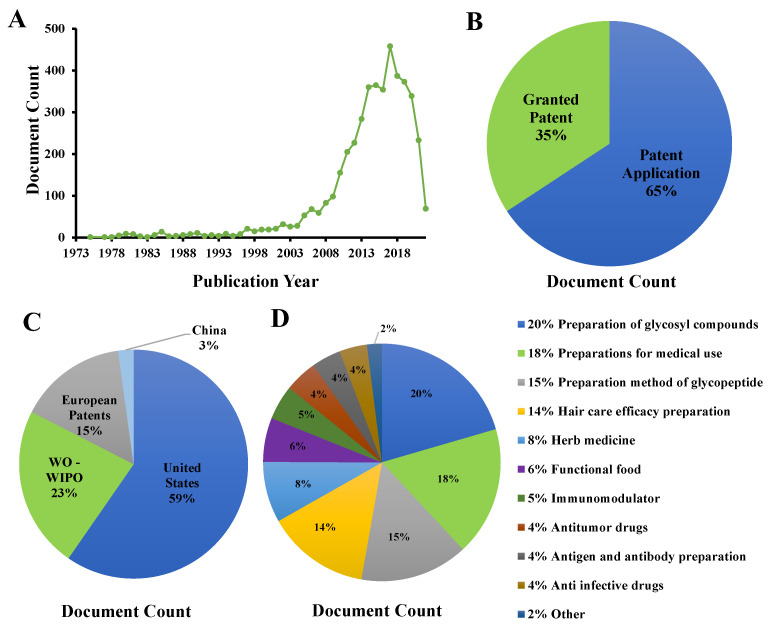
Analysis of 4441 patents browsed in ww.lens.org using the search terms “*Coriolus Versicolor* polysaccharide”: (**A**) patent applications per years; (**B**) document type; (**C**) jurisdictions (WO-WIPO, world intellectual property organization); (**D**) central product classification.

In the past decades, polysaccharides have attracted more and more attention. Many natural polysaccharides have been developed for modern food supplements and clinical drugs [84,85]. CVP has great application potential in product development because of its natural, green, and excellent biological activity. Figure 4 provides a detailed analysis of the patent status of CVP. At present, there are 4441 CVP-related patents in the world. The largest proportion of patent applications is 65% and the smallest proportion of patent authorization is 35%; but, there are very few research reports. Among them, the United States and the world intellectual property organization have the most patents, accounting for 82% of the total, while China and other countries have a small proportion, accounting for only 3%. The patents related to CVP mainly focus on the extraction method of CVP, while the application products of bioactivity account for less. Generally speaking, the application and development of CVP are still in the primary stage. Although there are some research and development of functional foods, functional beverages, and drugs, there are still problems such as small quantity, single application field, and less development of active species. In future research, we need to strengthen the research on this aspect and give full play to the application potential of CVP.

## 6. Conclusions and Perspectives

At present, the most widely used extraction method of CVP is hot water extraction, with an extraction rate of 5–7%; it uses simple equipment, is a simple method, and allows safety and environmental protection. On this basis, the extraction process of CVP is improved by enzyme extraction, ultrasonic extraction, and other auxiliary means, which can greatly shorten the extraction time, and reduce the particle size of the raw materials by liquid nitrogen grinding and other methods, which can significantly improve the extraction rate. In addition, the structure analysis of CVP shows that its monosaccharide is mainly glucose, and some components also contained a small number of Man, Rha, Gal, and Fuc. The main chain is composed of the (1→4)-β-/(1→3)-β-d-glucopyranosyl group, and branches attached to the O-6 site. At the same time, it has many biological activities, such as antioxidant, antitumor, and immune regulation. It can be applied to health food, and its new biological activities need to be explored.

Although the development of CVP and related scientific research have made great progress, there are still some problems. Some suggestions are also put forward for these problems. The main ones are as follows: (1) The exploration scope of the CVP extraction process is not wide enough and the innovation is poor, which mainly stays in the research of traditional extraction methods. Therefore, it is necessary to explore new extraction methods of CVP and try to connect with new extraction methods, such as supercritical fluid extraction and ultrafiltration extraction, so as to make up for the shortcomings of the existing extraction methods. (2) The depth of structural analysis of CVP is not enough. At present, the research on the structure of CVP mainly focuses on its primary structure, and there is a lack of research on its advanced structure, which is not conducive to the research on the structure–activity relationship of CVP. Therefore, it is necessary to use advanced atomic force microscopy (AFM), X-ray diffraction (XRD), circular dichroism (CD), and other technical means to study the advanced structure of CVP. (3) Many biological activities of CVP have not been linked with practical application. At present, there are 156 kinds of drugs and functional foods, but they are related to liver protection, and other biological activities have not been applied. Therefore, it is necessary to give full play to the practical application of other biological activities of CVP and broaden its application range. In general, CVP has great development potential and application value, which is worthy of further research in extraction, separation and purification, structural characterization, and biological activity.

## Figures and Tables

**Table 1:** Extraction methods and yields of CVP.

Experimental Method	Temperature	Time	Other Conditions	Yields	Ref.
Hot water extraction	90 °C	120 min	Material-to-liquid ratio of 1:30 (g:mL)	5.38%	[14]
	100 °C	120 min	Ethanol concentration 95%	6.98%	[15]
	85 °C	105 min	Feed-to-liquid ratio of 1:27	7.27%	[16]
	100 °C	180 min	Feed-to-liquid ratio of 1:50, 3 h	4.39%	[17]
	80 °C	180 min	Liquid nitrogen grinding	16.1%	[18]
Enzyme extraction	52 °C	37 min	pH 5.5, enzyme concentration: 2.50%	9.58%	[19]
	55 °C	20 min	pH 6.0, enzyme concentration: 1.50%	3.26%	[20]
Ultrasonic extraction	Room temperature	15 min	Material-to-liquid ratio of 1:20 (g:mL), ultrasonic extraction twice	3.84%	[21]
	45 °C	50 min	pH 8.5, 2% Na_2_CO_3_	13.87%	[22]
	Room temperature	30 min	Material-to-liquid ratio of 1:45 (g:mL), ultrasonic power 450 W	13.6%	[23]

**Table 2:** CVP antioxidant activity index and research results.

Vitro/Vivo	Effect	Ref.
In vitro	The scavenging activity of DPPH radical (64.9% at 0.8 mg/mL), O_2•−_ radical (78.4% at 1.2 mg/mL) and OH radical (71.2% at 2.0 mg/mL)	[9]
	IC_50_ (DPPH radical) = 0.388 mg/mL, IC_50_ (ABTS radical) = 0.419 mg/mL, IC_50_ (OH radical) = 4.423 mg/mL	[43]
	The best scavenging activity of O_2•_^−^ (60% at 5 mg/mL), OH radical (81% at 5 mg/mL)	[44]
	IC_50_ = 0.832 g/mL	[45]
	The best scavenging activity of ABTS radical (53% at 10 mg/mL), OH radical (72% at 5 mg/mL)	[46]
In vivo	SOD↑, GSH-Px↑, MDA↓	[47]
	SOD↑, CAT↑	[48]
	Ox-LDL↓, SOD↑	[49]

Notes: ↑, Increased; ↓, reduced.

## Data Availability

No new data were created or analyzed in this study. Data sharing is not applicable to this article.
